# Commentary on Eichinger J, Zimmermann B, Elger B, McLennan S, Filges I, Koné I. 2023. ‘It’s a nightmare’: informed consent in paediatric genome-wide sequencing. A qualitative expert interview study from Germany and Switzerland

**DOI:** 10.1038/s41431-023-01490-x

**Published:** 2023-11-10

**Authors:** Angus J. Clarke

**Affiliations:** School of Medicine, Cardiff University, Wales UK

**Keywords:** Genetic testing, Paediatrics, Medical ethics

This paper, reporting the views of medical genetics practitioners from Germany and Switzerland about the consent process for genome-based investigations of children, is timely. It shows the substantial challenges in clinical practice and, in particular, demonstrates the diversity of opinions among practitioners.

One of these challenges is the need to obtain parental consent before proceeding with whole genome sequencing (WGS). ‘Consent’ does not really need the qualifier ‘informed’, as it is not valid if the person ‘giving’ consent does not understand what they are agreeing to, but the two words seem almost to have become inseparable in the context of genetic testing. This is unfortunate as it perhaps accounts for the undue weight that we clinical geneticists - as a profession - have often given to the provision of information before genetic testing, as opposed to other relevant factors that we may need to address.

Because of these other constraints, the agreement of parents to a practitioner’s suggestion for their child to have WGS may not be valid for other reasons too. Thus, the parent may feel swamped by an excess of technical information - too much information, as opposed to too little - or they may feel coerced by the expectation of the practitioner, or they may be desperate to do ‘anything’ for the sake of their child, so that they fail to reflect and deliberate effectively about the decision.

The verbal couplet ‘informed consent’ does not lead us to question whether we are giving too much information, or might be perceived by the parent as being coercive, or whether the parent is in a suitable frame of mind to make their decision. Perhaps it would be better if we dropped the word ‘informed’ as it has distracted us so effectively from these other, equally important potential difficulties with the whole process of, ‘information, explanation and consent’.

What is key is for parents to understand the types of results that may emerge from testing and some of the limitations and implications of genome-based investigations. This applies especially to WGS but also other genome-based tests, such as exome analysis, dosage analysis and even karyotype. The paper by Eichinger et al is most helpful in drawing our attention to many of these points.

The types of results include:

I. A clear result that explains the child’s condition, and that will sometimes lead to a specific, established treatment, but may also fail to bring such benefits. It may permit a more accurate prognosis, and it may have reproductive implications for the parents, the child him/her-self or other members of the family, but it may not.
II. No result of relevance to the child’s condition.
III. A variant of uncertain significance (VUS) may be found, in a gene in which other variants could account for the child’s condition. It may take time for the VUS to be interpreted as pathogenic or benign; samples from other members of the family will sometimes be helpful in that process.
IV. Other genetic information, unrelated to the child’s current difficulties but of potential future medical importance, will sometimes also be relevant to other members of the family. Parents may be encouraged to defer making decisions about whether to access secondary findings, and what categories of secondary findings they would wish to access. This may be an especially helpful deferral of decision-making when the child is receiving neonatal or paediatric intensive care.
  The limitations and implications of genomic results:
V. Results may give probabilistic information about conditions of only modest penetrance or about the chance of disease complications. Such information arises especially in familial cancer disorders.
VI. The provisional nature of much genomic diagnostics may be mentioned, so that parents are aware of the potential for a change in interpretation of genomic results over time (this does not only apply to a VUS).
VII. Consent for some genetic research and investigations in childhood may need to be renewed by the ‘child’ once they become mature or adult, if they have capacity.
VIII. It may be helpful to remind parents that the findings of genome-based testing are likely to entail clinical and/or reproductive implications for the child (once adult or mature) and other relatives. Attention therefore needs to be paid to when and how these implications will be raised with the ‘child’ and other members of the family.

Conveying these points to parents is not usually difficult. No mastery of technical information is required. It may be more difficult to counter obstacles to understanding, such as excessively high expectations of the benefits to come from genetic testing or preconceptions about the origin of the child’s problem. Such obstacles will need to be addressed, with appropriate sensitivity [1].

Where a diagnosis is sought for a sick child, the question of non-directiveness should hardly arise: appropriate investigation can be whole-heartedly recommended. The decision of course rests with the parents but the practitioner can hardly be neutral (“indifferent”) to what is decided. The context is very different in the areas of reproduction (prenatal decisions and carrier testing) and predictive testing for untreatable diseases, such as many of the adult-onset neurodegenerative disorders. In those contexts, the principle of non-directiveness requires as much respect as ever.

The very different perspectives found among the interviewees in the study by Eichinger et al is notable. Some clinicians feel the need for formalised consent that entails the transfer of quantities of information. Others are concerned to ensure that parents understand what is at stake, rather than the masses of detail. What can we do to encourage the latter approach, favouring quality, and help the former group of clinicians to ‘relax’ their focus on quantity?

One approach would be to address the question of insufficient practitioner time. This leads us to the need for genetic counsellors to be recognised as a professional group in those European countries that have not yet accepted it. This applies not only to Germany and Switzerland but several other countries too. For German-speaking countries, a good start has been made with the excellent MSc course in Genetic & Genomic Counselling, now well established in Innsbruck.

However, the simplest and perhaps the most important step we can take towards improving the quality of consent in genomic testing is to lose the word ‘informed’ and talk simply of ‘consent’.
